# Genome-wide characterization of the AP2/ERF gene family in *Scutellaria baicalensis* Georgi reveals root-preferential and MeJA-responsive candidate genes

**DOI:** 10.3389/fpls.2026.1971990

**Published:** 2026-09-17

**Authors:** Chaoyi Li, Yang Lu, Chunying Zhao, Xiangling Liu

**Affiliations:** Hebei Province Key Laboratory of Study and Exploitation of Chinese Medicine, Institute of Chinese Materia Medica, Chengde Medical University, Chengde, China

**Keywords:** AP2/ERF transcription factors, flavone biosynthesis, methyl jasmonate, root-preferential expression, *Scutellaria baicalensis*

## Abstract

APETALA2/ethylene-responsive factor (AP2/ERF) transcription factors play central roles in plant growth, development and environmental responses. *Scutellaria baicalensis* is a medicinal plant that predominantly accumulates pharmacologically important flavones in its roots, and methyl jasmonate (MeJA) induced these flavones accumulation. However, the AP2/ERF family has not been systematically characterized in *S*. *baicalensis*, and members showing root-preferential expression and MeJA responsiveness remain unknown. Here, we identified 137 *SbAP2/ERF* genes at the genome-wide level and investigated their phylogenetic relationships, conserved motifs, domain architectures, gene structures, chromosomal distribution, duplication patterns, interspecific collinearity, promoter cis-acting elements, and expression profiles. The identified 137 genes were classified into the ERF (68), DREB (40), AP2 (24), RAV (3), and Soloist (2) subfamilies. Most members within each subfamily shared similar structural features, whereas marked differences were observed among subfamilies. Of the 63 nonredundant duplicated gene pairs, 54 were segmental and nine were tandem duplicates. Promoter analysis identified 4,491 predicted cis-acting element sites associated mainly with light, hormone, and stress responses, and MeJA-responsive motifs co-occurred in 95 promoters. Analysis of publicly available RNA sequencing (RNA-seq) datasets revealed diverse organ-preferential expression patterns and time-dependent transcriptional responses to MeJA. Combining the RNA-seq profiles and qRT-PCR measurements identified eight candidate genes which were preferentially expressed in roots and significantly upregulated by MeJA including *SbAP2-13*, *SbAP2-14*, *SbAP2-27*, *SbAP2-40*, *SbAP2-59*, *SbAP2-117*, *SbAP2-130*, and *SbAP2-137*. These results provide a comprehensive resource for the SbAP2/ERF family and prioritize candidate genes for investigating MeJA-responsive transcriptional processes and the potential regulation of root-specific flavones biosynthesis in *S*. *baicalensis*.

## 1 Introduction

*Scutellaria baicalensis* Georgi (*S*. *baicalensis*) is an important traditional medicinal plant belonging to the Lamiaceae family. Its dried roots are widely used as a traditional Chinese medicinal material. Baicalin and other flavones are the major bioactive constituents of *S*. *baicalensis* and have been reported to exhibit anti-inflammatory, antiviral, and antioxidant activities (Zhao et al., 2016a, 2016b). With the availability of genomic and transcriptomic resources for *S*. *baicalensis*, the biosynthetic pathways of its flavones have been progressively elucidated. Two distinct pathways have been identified. The classical pathway primarily produces the 4′-hydroxyflavones scutellarein and scutellarin in aerial tissues, whereas the root-specific flavones pathway produces 4′-deoxyflavones, including baicalein, wogonin, baicalin, and wogonoside. Although considerable progress has been made in elucidating these pathways, their transcriptional regulation remains insufficiently understood.

Transcription factors are key components of gene regulatory networks and control or affect various biological processes by activating or repressing the expression of specific target genes. The APETALA2/ethylene-responsive factor (AP2/ERF) family is one of the largest plant-specific transcription factor families and has been systematically identified in various plant species, including *Arabidopsis thaliana* (*A*. *thaliana*), *Oryza sativa* (*O*. *sativa*), *Juglans mandshurica*, *Elaeis guineensis*, and *Lonicera japonica* (Nakano et al., 2006; Zhou and Yarra, 2021; Zhao et al., 2022; Li et al., 2026). AP2/ERF family members contain at least one highly conserved AP2 DNA-binding domain comprising approximately 60–70 amino acid residues. Based on the number and sequence characteristics of AP2 domains, the AP2/ERF proteins are generally classified into five subfamilies: AP2, ERF, DREB, RAV, and Soloist. Members of the AP2 subfamily usually contain two AP2 domains, whereas ERF and DREB proteins generally contain a single AP2 domain. RAV proteins possess both an AP2 domain and a B3 domain, while Soloist proteins are classified separately because of their unique structural features (Licausi et al., 2010). AP2/ERF transcription factors are widely involved in plant growth and development, hormone signal transduction, and responses to environmental stresses. Many AP2 subfamily members participate in organogenesis and developmental regulation (Xu et al., 2013; Liu et al., 2023), DREB members are commonly associated with responses to abiotic stresses (Wang et al., 2024), and numerous ERF members function in hormone signaling and plant defense responses (Lee et al., 2016; Tang et al., 2025).

AP2/ERF transcription factors play important roles in the regulation of secondary metabolism in medicinal plants. Specific AP2/ERF members have been reported to regulate the biosynthesis of terpenoids, flavonoids, and other specialized metabolites. AP2/ERF transcription factors were reported to increase the accumulation of target terpenoid compounds in *Artemisia annua*, *Zea mays* (*Z*. *mays*), and *Litsea cubeba* (Yu et al., 2012; Li et al., 2015; Wang et al., 2022). In addition, overexpression of *CtERF_6*, *CtERF_32* or *CtDREB_15* could significantly promote flavonoid accumulation in *Carthamus tinctorius* (Hu et al., 2026). The *CmERF/PTI6* gene has been proposed as a potential upstream regulator involved in the biosynthesis of medicinal secondary metabolites, including flavonoids and caffeoylquinic acids in *Chrysanthemum morifolium* (Lu et al., 2024). *FhAP2* and *LhERF4* negatively affect anthocyanin biosynthesis through distinct MYB-related pathways in *Freesia hybrida* and *Lilium* spp. respectively (Cao et al., 2024; Zhang et al., 2026). In citrus, three AP2/ERF transcription factors were shown to promote flavonoid biosynthesis by activating *CitCHIL1* (Zhao et al., 2021). Moreover, AP2/ERF family members were involved in regulating the biosynthesis of other secondary metabolites, including tanshinones in *Salvia miltiorrhiza* and β-patchoulene in *Dendrobium officinale* (Li et al., 2024a, 2024b).

Despite these advances, a comprehensive genome-wide characterization of the AP2/ERF family in *S*. *baicalensis* remains lacking, and the family members potentially associated with flavone biosynthesis have not been investigated. In this study, we systematically identified AP2/ERF family members in the *S*. *baicalensis* genome and characterized their evolutionary, structural, genomic, and promoter features. Publicly available RNA-seq datasets were reanalyzed to characterize the expression patterns of *SbAP2/ERF* genes across different organs and under methyl jasmonate (MeJA) treatment. By integrating evidence of RNA-seq and quantitative real-time PCR (qRT-PCR) validation, candidate genes potentially associated with MeJA responsiveness and root-preferential expression were identified. This study provided important clues for investigating the potential roles of *SbAP2/ERF* genes in the transcriptional regulation of MeJA responses and root-specific flavones biosynthesis in *S*. *baicalensis* roots.

## 2 Materials and methods

### 2.1 Genome-wide identification of AP2/ERF family members in *S*. *baicalensis*

The genome sequence, protein sequence, and genome annotation files of *S*. *baicalensis* (PRJCA016803) were downloaded from the National Genomics Data Center (NGDC, https://www.cncb.ac.cn/). The AP2/ERF protein sequences of *A*. *thaliana* were obtained from The Arabidopsis Information Resource (TAIR, https://www.arabidopsis.org). The AP2/ERF family members in *S*. *baicalensis* were identified using a combined BLASTP and Hidden Markov Model (HMM) search strategy. The AP2/ERF protein sequences of *A*. *thaliana* were used as query sequences to search against the *S*. *baicalensis* protein database using the BLASTP module in TBtools-II v2.485 (Chen et al., 2023), with an E-value threshold of 1e-5. Additionally, the HMM profile of the AP2 domain (PF00847) was downloaded from the Pfam database and used to screen the *S*. *baicalensis* protein sequences. Based on the candidate sequences obtained from the initial screening, a species-specific HMM profile was constructed and used to rescreen the complete *S*. *baicalensis* protein dataset, with an E-value threshold of 1e-5. The candidate sequences obtained from BLASTP and HMM-based searches were pooled and deduplicated. Conserved domains in the resulting proteins were examined using the NCBI Batch Conserved Domain Search (https://www.ncbi.nlm.nih.gov/Structure/cdd/wrpsb.cgi). The candidate proteins were further cross validated using InterProScan v5.78-109.0, and evidence from CDD, Pfam, SMART, PROSITE, and Gene3D was considered collectively (Jones et al., 2014). A total of 137 putative AP2/ERF family members in *S*. *baicalensis* were identified and were designated SbAP2–1 to SbAP2–137 according to their chromosomal positions. Here, the prefix “SbAP2” is used for all members of the SbAP2/ERF family and does not indicate assignment to the AP2 subfamily.

### 2.2 Analysis of physicochemical properties, subcellular localization, and transmembrane domains

The physicochemical properties of SbAP2/ERF proteins, including amino acid length, molecular weight, theoretical isoelectric point (pI), instability index, and aliphatic index, were analyzed using the ExPASy ProtParam tool (https://web.expasy.org/protparam/). Subcellular localization of SbAP2/ERF proteins was predicted using WoLF PSORT (https://wolfpsort.hgc.jp/), and transmembrane domains were predicted using TMHMM (https://services.healthtech.dtu.dk/services/TMHMM-2.0/).

### 2.3 Sequence alignment and phylogenetic analysis

Full-length AP2/ERF amino acid sequences from *A*. *thaliana* and *S*. *baicalensis* were combined, and terminal stop symbols were removed before phylogenetic analysis. The protein sequences were aligned using MAFFT v7.526 with the L-INS-i algorithm, incorporating local pairwise alignment and 1,000 iterative refinement cycles (Katoh and Standley, 2013). Alignment columns containing more than 50% gaps or ambiguous residues were removed using the gappy mode implemented in ClipKIT v2.13.1 (Steenwyk et al., 2020). A maximum-likelihood phylogeny was subsequently constructed from the filtered alignment using IQ-TREE v3.1.3 (Minh et al., 2020). ModelFinder was used to select the best-fitting amino acid substitution model according to the Bayesian information criterion (Kalyaanamoorthy et al., 2017). Branch support was assessed using 1,000 replicates of the SH-like approximate likelihood ratio test and 1,000 ultrafast bootstrap replicates. Bootstrap nearest-neighbor interchange optimization was enabled (Hoang et al., 2018).

### 2.4 Conserved motif, domain, and gene structure analysis

Conserved motifs in the SbAP2/ERF protein sequences were identified using MEME Suite (https://meme-suite.org/), with the maximum number of motifs set to ten. Conserved domains were identified using the NCBI Batch Conserved Domain Search, and the domain types and their start and end positions within each protein sequence were extracted. Based on the *S*. *baicalensis* genome annotation file, the exon–intron structures of the *SbAP2/ERF* genes were analyzed and visualized using TBtools-II.

### 2.5 Chromosomal distribution, gene duplication, and interspecific collinearity analysis

The chromosomal locations of the *SbAP2/ERF* genes were obtained from the *S*. *baicalensis* genome annotation (GWHDEDD00000000). The chromosomal distribution was visualized according to the physical coordinates. Based on the *S*. *baicalensis* reference genome annotation file, an all-versus-all protein similarity search was performed using DIAMOND v2.2.1 in sensitive mode. Intragenomic collinear blocks and duplicate genes were identified using MCScanX v1.0.0 with a match score of 50, a minimum of five anchors per collinear block, a maximum gap of 25 genes, and a block significance threshold of 1e-5 by integrating the protein similarity results with genomic coordinates (Wang et al., 2012). A pair was classified as a segmental duplicate when both genes belonged to the 137 SbAP2/ERF members and formed a direct anchor pair in the MCScanX collinearity output. Pairs of SbAP2/ERF members reported in the MCScanX tandem output were classified as tandem duplicates. All gene pairs were treated as undirected relationships and deduplicated before counting. Gene density was calculated using non-overlapping 200-kb windows. Matplotlib v3.11.1 was used to visualize gene positions, gene-density tracks, genome-wide collinear blocks, and *SbAP2/ERF* duplication relationships. Protein sequences of each duplicate gene pair were aligned using MAFFT v7.526. The resulting alignments were back-translated into codon alignments based on the corresponding coding sequences. Codon columns containing gaps in either sequence were removed. Ka, Ks, and Ka/Ks ratios were subsequently estimated using the yn00 program implemented in PAML v4.10.10 under the Yang–Nielsen method (Yang and Nielsen, 2000; Yang, 2007). Gene pairs with Ks ≤ 0 or the sentinel value Ks = 99 returned by yn00 were considered unestimable. Gene pairs with Ks > 3 were considered unreliable for selection pressure inference. Collinear relationships between *S*. *baicalensis* and *A*. *thaliana*, *Scutellaria barbata* (*S*. *barbata*), *Populus trichocarpa* (*P*. *trichocarpa*), *Solanum lycopersicum* (*S*. *lycopersicum*), *Solanum tuberosum* (*S*. *tuberosum*), *O*. *sativa*, and *Z*. *mays*, were analyzed using the One Step MCScanX–Super Fast module in TBtools-II. Pairwise comparisons were performed between *S*. *baicalensis* and seven plant species. BLASTP searches were conducted using two CPU threads with an E-value threshold of 1e-3, and the top ten hits were retained for each query sequence. Collinear blocks were subsequently identified using MCScanX. The distribution of homologous gene pairs was visualized using TBtools-II.

### 2.6 Cis-acting element analysis in *SbAP2/ERF* promoters

Based on the *S*. *baicalensis* genome sequence and corresponding GFF3 annotation, the 2,000-bp genomic region upstream of the annotated transcription start site of each *SbAP2/ERF* gene was extracted as putative promoter region. These sequences were submitted to the PlantCARE database (http://bioinformatics.psb.ugent.be/webtools/plantcare/html/) to predict cis-acting elements (Lescot et al., 2002). The PlantCARE outputs were processed and visualized using Python.

### 2.7 Transcriptome-based expression pattern analysis

RNA-seq data from different organs of *S*. *baicalensis*, including roots, stems, leaves, and petals (PRJNA1079421, PRJNA515574), as well as RNA-seq data from root samples with/without MeJA treatment (PRJNA961700, PRJNA485226), were downloaded from the NCBI public database (Gao et al., 2019; Zhao et al., 2019; Fang Y. et al., 2023; Yao et al., 2024). The corresponding SRA Run data within each BioProject were imported into TBtools-II and converted into FASTQ files using the Convert SRA to Fastq Files function. The FASTQ files were further analyzed using the kallisto RNA-seq quantification program through the Kallisto Super Wrapper in TBtools-II. The RNA sequence file corresponding to the GWHDEDD00000000 reference genome assembly was used as the reference transcript set. Transcript abundance was quantified and expressed as transcripts per million (TPM) (Bray et al., 2016). Based on the TPM values, expression heatmaps were generated to analyze the expression patterns of *SbAP2/ERF* genes in different organs and under MeJA treatment. The count matrix generated by kallisto was used as input for differential expression analysis with the DESeq2 module of Batch DEGs Analysis Tools in TBtools-II. Genes with |log2 fold change (FC)| ≥ 1 and adjusted *p* value (*p* adj) < 0.05 were considered significantly differentially expressed. Datasets from different studies were analyzed separately and were not merged across datasets. All group comparisons were performed within the corresponding datasets according to their respective experimental designs.

### 2.8 Plant materials, hormone treatment, and sample collection

Seeds of the *S. baicalensis* cultivar “Jiqin No. 1” were collected in Chengde, China, and sown in a 3:1:1 mixture of peat moss-enriched soil, vermiculite, and perlite. The seeds were cultivated under a 16/8 h light/dark photoperiod (light level of 12000 lux) at 23 °C. One-month-old *S. baicalensis* plants were used for MeJA treatment. Plants were sprayed with 100 μM MeJA solution or distilled water. Root samples were collected 24 h after treatment and further used for qRT-PCR analysis. Roots, stems, leaves, and petals were collected from four-month-old *S. baicalensis* plants with uniform growth at the flowering stage and further used for qRT-PCR analysis of different organs. And three independently collected replicates were analyzed per treatment or organ.

### 2.9 RNA extraction and qRT-PCR analysis

Total RNA was extracted from each sample using the E.Z.N.A. Plant RNA Kit (OMEGA: R6827). Genomic DNA was removed, and first-strand cDNA was synthesized using a TransGen reverse transcription kit (AU341). The qRT-PCR was performed using a CFX Connect Real-Time PCR Detection System with a 96-well format (Bio-Rad, USA). The amplification program was as follows: pre-denaturation at 94 °C for 30 s, followed by 40 cycles of denaturation at 94 °C for 5 s and annealing/extension at 60 °C for 30 s. Melting curve analysis was performed after amplification to verify the specificity of PCR products. According to a previous study in *S*. *baicalensis*, *ACT7* showed the highest stability under hormone stimuli treatments, whereas *UBC* was one of the most stable reference genes (Wang et al., 2021). Accordingly, *ACT7* was used as the internal control for the MeJA treatment assays, whereas *UBC* was used as reference gene for different organs in this study. The relative expression level was calculated by the 2^−ΔΔCt^ method. All primers were listed in **Supplementary Table 1**.

### 2.10 Statistical analysis

All experiments included three biological replicates. The data were presented as the mean ± standard deviation. Statistical analyses were performed using GraphPad Prism v10.1.2. Differences in gene expression among different organs were analyzed using one-way analysis of variance followed by Tukey’s multiple comparison test. Differences between MeJA-treated and control samples were analyzed using Student’s *t*-test. Differences were considered statistically significant at *p* < 0.05.

### 2.11 Accession numbers

The *S. baicalensis* genome used in this study is publicly available through the NGDC under BioProject accession PRJCA016803 and GWH accession GWHDEDD00000000. Public RNA-seq datasets analyzed in this work are accessible through NCBI under BioProject accessions PRJNA1079421, PRJNA515574, PRJNA961700, and PRJNA485226.

## 3 Results

### 3.1 Identification and physicochemical characterization of the SbAP2/ERF family

A total of 137 nonredundant AP2/ERF family members were identified in the *S*. *baicalensis* genome through a combination of BLASTP, HMMER, NCBI CD-Search, and InterProScan analysis. These genes were named *SbAP2–1* to *SbAP2–137* according to their chromosomal positions. The encoded SbAP2/ERF proteins ranged from 99 aa to 629 aa, with predicted molecular weights ranging from 11,153.52 Da to 69,010.83 Da, respectively. Among the 137 SbAP2/ERF proteins, nine had lengths of 150 aa or less, including SbAP2-126, the shortest member at 99 aa. All nine proteins passed quality control of the annotated gene models and AP2/ERF domain validation and were therefore retained rather than excluded solely because of their short lengths. The theoretical isoelectric points of the 137 SbAP2/ERF proteins ranged from 4.50 to 10.37, with a mean value of 6.90. Based on these values, 84 were predicted to be acidic (pI < 7), whereas 53 were predicted to be basic (pI > 7), indicating a predominance of acidic proteins within the family. The instability and aliphatic indices ranged from 25.08 to 75.90 and from 39.12 to 91.43, respectively (**Supplementary Table 2**). Overall, these physicochemical profiles reveal marked diversity in protein size, charge characteristics, predicted stability, and aliphatic amino acid composition among SbAP2/ERF family members.

Transmembrane domain prediction showed that most SbAP2/ERF proteins lacked predicted transmembrane regions. Subcellular localization analysis predicted that 112 of the 137 proteins (81.8%) were localized to the nucleus, consistent with their putative roles as transcriptional regulators. The remaining 25 proteins were assigned to chloroplasts (15), the cytoplasm (5), mitochondria (4), or both chloroplasts and mitochondria (1), suggesting that SbAP2/ERF family members exhibit different predicted subcellular localization patterns (**Supplementary Table 2**).

### 3.2 Phylogenetic analysis and classification of the SbAP2/ERF family

To investigate the phylogenetic relationships and classify the AP2/ERF family members in *S*. *baicalensis*, a maximum-likelihood phylogenetic tree was constructed using 137 SbAP2/ERF proteins and 147 AP2/ERF proteins from *A*. *thaliana* (**Figure 1**). Based on their positions relative to the previously classified *A*. *thaliana* reference proteins, the SbAP2/ERF proteins were assigned to five subfamilies: ERF, DREB, AP2, RAV, and Soloist. The ERF subfamily was the largest, comprising 68 members, followed by the DREB and AP2 subfamilies with 40 and 24 members, respectively. The RAV subfamily contained three members, whereas SbAP2–58 and SbAP2–132 clustered with the *A*. *thaliana* Soloist protein At4G13040 and were therefore assigned to the Soloist subfamily (**Figure 1**; **Supplementary Table 3**).

**Figure 1:**
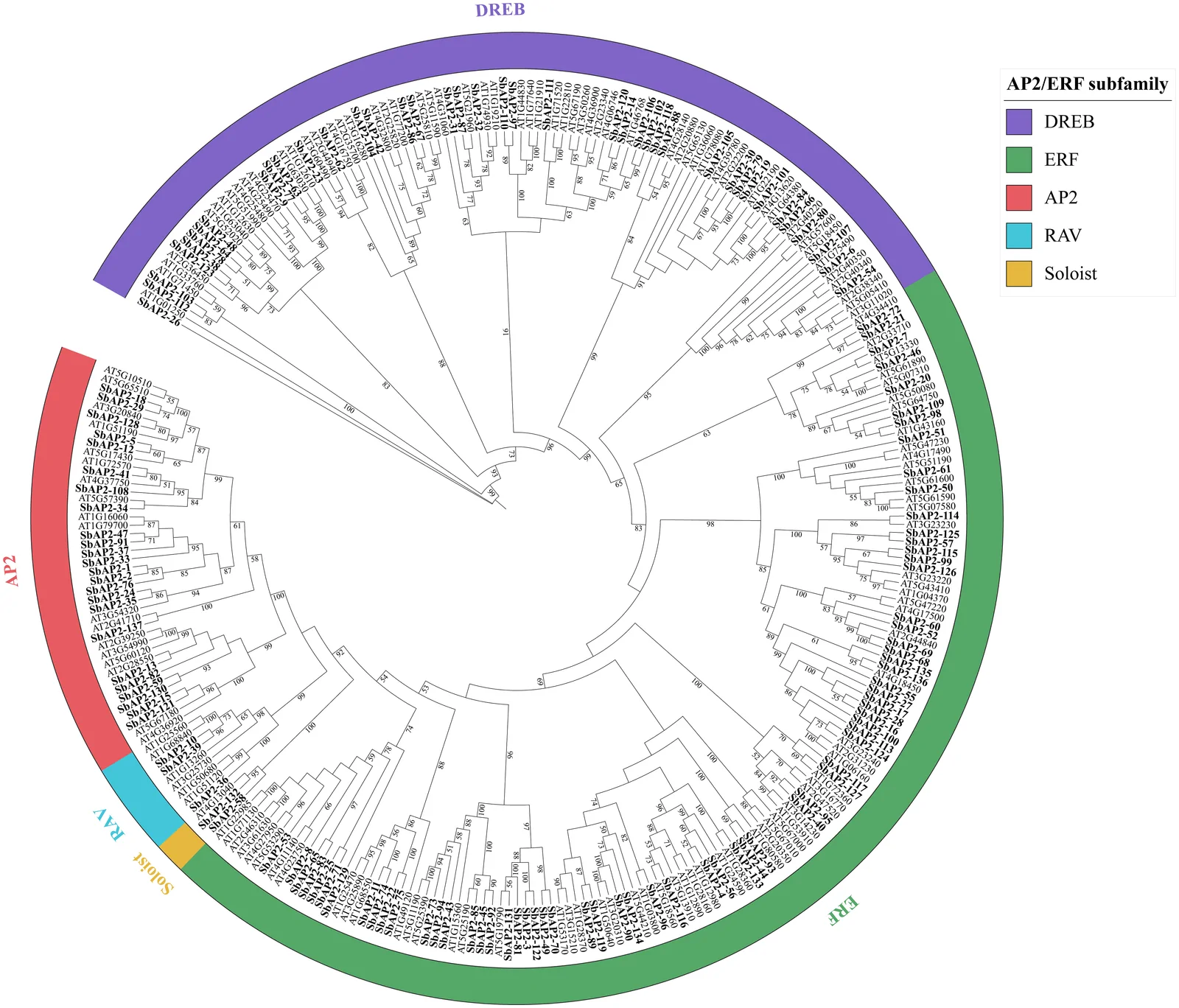
Phylogenetic relationships of AP2/ERF proteins from *S*. *baicalensis* and *A*. *thaliana*. The maximum-likelihood phylogenetic tree was constructed using 137 AP2/ERF protein sequences from *S*. *baicalensis* and 147 AP2/ERF protein sequences from *A*. *thaliana*. The colored outer sectors indicate the DREB, ERF, AP2, RAV, and Soloist subfamilies. SbAP2/ERF protein names are shown in bold, whereas *A*. *thaliana* protein names are shown in regular font. Node values indicate ultrafast bootstrap support (UFBoot), with only UFBoot values >50% displayed.

A total of 13 strongly supported SbAP2/ERF sister gene pairs were identified in the combined phylogeny (UFBoot ≥ 95%). The DREB subfamily contained four pairs: SbAP2-63/SbAP2-77 (UFBoot = 95%), SbAP2-102/SbAP2-106 (UFBoot = 99%), SbAP2-19/SbAP2-79 (UFBoot = 100%), and SbAP2-66/SbAP2-84 (UFBoot = 95%). The ERF subfamily contained eight pairs: SbAP2-22/SbAP2-75 (UFBoot = 100%), SbAP2-57/SbAP2-125 (UFBoot = 97%), SbAP2-68/SbAP2-69 (UFBoot = 100%), SbAP2-21/SbAP2-72 (UFBoot = 97%), SbAP2-65/SbAP2-83 (UFBoot = 97%), SbAP2-11/SbAP2-74 (UFBoot = 100%), SbAP2-3/SbAP2-122 (UFBoot = 100%), and SbAP2-49/SbAP2-70 (UFBoot = 98%). In addition, SbAP2–10 and SbAP2–39 formed a strongly supported sister pair in the RAV subfamily (UFBoot = 96%). No strongly supported SbAP2/ERF sister pairs were identified in the AP2 or Soloist subfamilies (**Figure 1**).

### 3.3 Conserved motifs, domain architectures, and gene structures of SbAP2/ERF members

To further characterize structural patterns within the SbAP2/ERF family, conserved motifs, domain architectures, and gene structures were analyzed in the 137 SbAP2/ERF members (**Figure 2**). The combined phylogenetic tree was pruned to retain only the 137 SbAP2/ERF proteins, which had been assigned to five subfamilies (**Figure 2A**). MEME analysis identified ten motifs, designated Motifs 1–10. The sequence logos revealed amino acid positions with varying levels of information content across the ten motifs (**Figure 2E**). Motif 1 was the most widely distributed and occurred in 136 of the 137 proteins, whereas Motifs 2 and 3 were each detected in 128 proteins and Motif 4 was present in 119 proteins. Most DREB and ERF proteins exhibited the motif arrangement Motif 2–Motif 4–Motif 1–Motif 3. Motif 8 occurred in 23 of the 40 DREB proteins, whereas Motif 10 was mainly detected in the ERF subfamily, occurring in 12 of its 68 members. AP2 proteins exhibited more complex motif architectures, with individual proteins containing three to seven motifs. Motifs 5, 6, and 7 occurred in 16, 22, and 15 of the 24 AP2 proteins, respectively. By contrast, the two Soloist proteins shared the relatively simple Motif 4–Motif 1 arrangement (**Figure 2B**; **Supplementary Table 4**).

**Figure 2:**
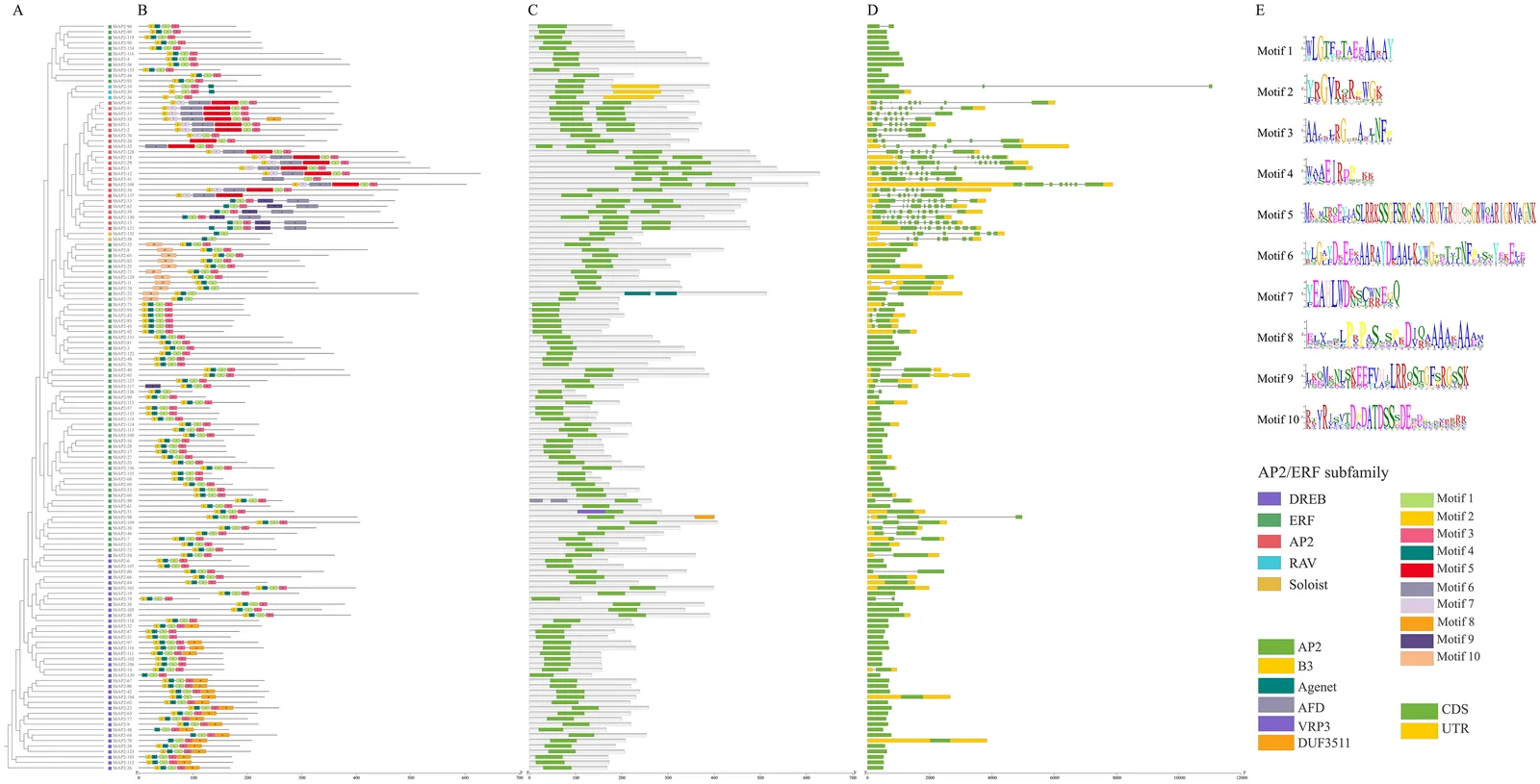
Phylogenetic relationships, conserved motif distributions, domain architectures, and gene structures of SbAP2/ERF family members. **(A)** Phylogenetic classification of SbAP2/ERF proteins. Colored squares indicate the DREB, ERF, AP2, RAV, and Soloist subfamilies, as shown in the legend. **(B)** Distribution of ten motifs in SbAP2/ERF proteins. Differently colored and numbered boxes represent Motifs 1–10, and gray lines represent the corresponding protein sequences. **(C)** Distribution of conserved domains in the SbAP2/ERF proteins. Differently colored boxes represent the domain annotations indicated in the legend, and gray bars represent the corresponding protein sequences. **(D)** Gene structures of the SbAP2/ERF members. Yellow and green boxes represent untranslated regions (UTRs) and coding sequences (CDSs), respectively, while the connecting lines represent introns. **(E)** Sequence logos of Motifs 1–10. The height of each amino acid indicates its relative conservation at the corresponding position, and the vertical axis represents information content in bits.

InterProScan detected the AP2/ERF domain (IPR001471) in all 137 members. Among them, 116 proteins contained a single AP2/ERF domain, whereas 21 contained two non-overlapping AP2/ERF domains. Members of the DREB, ERF, and Soloist subfamilies each contained a single AP2/ERF domain. All three RAV proteins possessed both an AP2/ERF domain and a B3 DNA-binding domain, consistent with the characteristic domain architecture of the RAV subfamily. Among the 24 AP2 subfamily members, 21 displayed the typical tandem AP2-domain architecture, whereas SbAP2-24, SbAP2-76, and SbAP2–137 each contained one confidently detected AP2/ERF region. To assess whether this pattern resulted from incomplete annotation, the gene models and CDS sequences were examined. All three genes showed complete coding regions with in-frame start and stop codons and no obvious premature truncation. In the phylogenetic tree, SbAP2–24 clustered with SbAP2-35 (UFBoot = 86%), SbAP2–76 clustered with SbAP2–1 and SbAP2-2 (UFBoot = 85%), and SbAP2–137 formed a sister relationship with At2G41710 (UFBoot = 100%) (**Figure 1**). Taken together, these results indicated that these proteins might be considered provisional atypical AP2 candidates (**Figure 2**). Additional domains were detected in a limited number of proteins, including Agenet-related domains in SbAP2–22 and a DUF3511 domain in SbAP2-98 (**Figure 2C**; **Supplementary Table 4**).

Gene structure analysis revealed distinct patterns among the five subfamilies. The number of exons per gene ranged from one to ten. Among the 137 putative *SbAP2/ERF* genes, 89 contained a single exon, whereas 48 contained multiple exons. Most *DREB* genes (38/40) and most *ERF* genes (49/68) contained a single exon, indicating relatively simple CDS organization. In contrast, *AP2* genes contained three to ten exons, and both *Soloist* genes contained six exons. The three *RAV* genes contained one to three exons (**Figure 2D**). Overall, these subfamily-associated patterns of motif organization, domain architecture, and gene structure were generally consistent with the phylogenetic classification of the SbAP2/ERF family and indicated greater structural similarity within subfamilies than among subfamilies.

### 3.4 Chromosomal distribution, gene duplication, and interspecific collinearity analysis of the *SbAP2/ERF* gene family

The 137 *SbAP2/ERF* genes were unevenly distributed across nine chromosomes (Chr1–Chr9) of *S*. *baicalensis.* Chr2 harbored the largest number of *SbAP2/ERF* genes, with 30 members accounting for 21.9% of the family, followed by Chr1, Chr9, Chr3, and Chr4, which contained 26, 18, 16, and 15 genes, respectively. Nine genes were located on Chr5, eight each on Chr6 and Chr7, and seven on Chr8. Together, Chr1 and Chr2 contained 56 genes, accounting for 40.9% of all identified *SbAP2/ERF* members (**Figure 3**; **Supplementary Table 3**).

**Figure 3:**
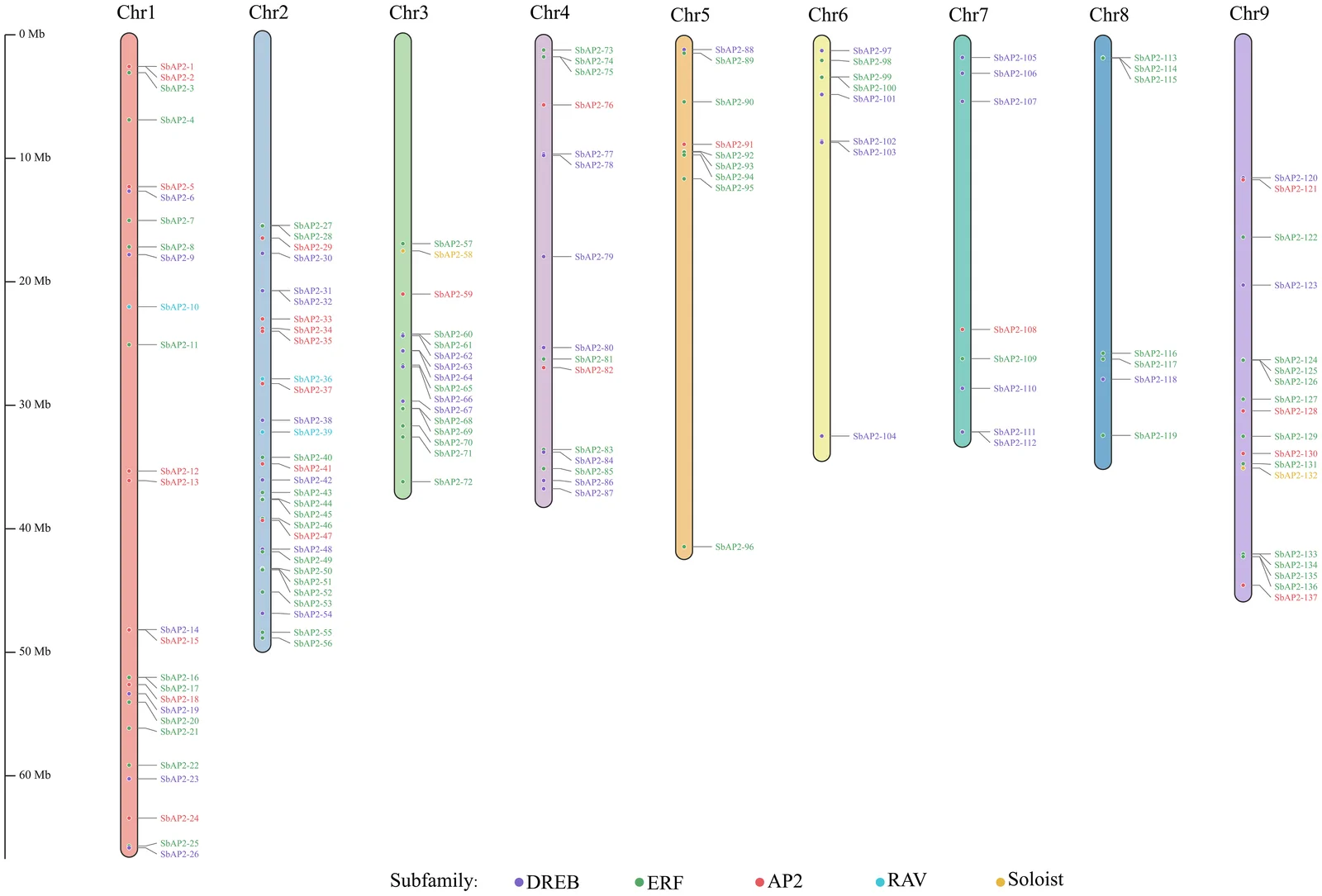
Chromosomal distribution of the *SbAP2/ERF* genes in *S*. *baicalensis*. A total of 137 *SbAP2/ERF* genes were mapped to chromosomes Chr1–Chr9 according to their physical positions. The scale on the left indicates chromosome length in megabases (Mb), and connecting lines indicate the positions of individual genes. Gene labels and position markers are colored according to their phylogenetically assigned subfamilies: DREB, purple; ERF, green; AP2, red; RAV, cyan; and Soloist, yellow.

The chromosomal distributions also differed among subfamilies. ERF, the largest subfamily, was distributed across all nine chromosomes, with 14 members on Chr2 and 11 members each on Chr1 and Chr9. DREB members were also distributed across all chromosomes, with seven members each on Chr2 and Chr4. AP2 members were concentrated mainly on Chr1 and Chr2, which contained eight and seven members, respectively, whereas no AP2 members were detected on Chr6 or Chr8. The three RAV members were located on Chr1 and Chr2, while the two Soloist members were located on Chr3 and Chr9 (**Figure 3**; **Supplementary Table 3**).

Intragenomic collinearity analysis identified 63 nonredundant *SbAP2/ERF* duplicate pairs, comprising 54 segmental and nine tandem duplicate pairs, which accounted for 85.7% and 14.3% of all detected duplication relationships, respectively (**Figure 4**). The segmental duplicate pairs involved 84 *SbAP2/ERF* genes, whereas the tandem duplicate pairs involved 17 *SbAP2/ERF* genes. After combining the two duplication categories and removing redundant genes, 98 *SbAP2/ERF* genes were involved in at least one duplication relationship, accounting for 71.5% of the family. Among them, *SbAP2-16*, *SbAP2-60*, and *SbAP2–133* participated in both segmental and tandem duplication relationships. Twenty-three genes were involved in at least two duplicate pairs. *SbAP2-25*, *SbAP2-65*, *SbAP2-71*, *SbAP2-119*, and *SbAP2–133* each occurred in three pairs. Genes involved in segmental duplication were distributed across all nine chromosomes. The nine tandem duplicate pairs were located on Chr1, Chr2, Chr3, Chr8, and Chr9, with three pairs on Chr2, two pairs each on Chr1 and Chr9, and one pair each on Chr3 and Chr8 (**Figure 4**; **Supplementary Table 5**). Together, these results suggested that segmental duplication played a greater role than tandem duplication in the expansion of the SbAP2/ERF family. In addition, Ka/Ks ratios were estimated for 61 of the 63 duplicate pairs, whereas the remaining two pairs were unestimable. Nineteen pairs with Ks > 3 were excluded from selection pressure inference because of potential synonymous site saturation (Shi and Chen, 2020). The remaining 42 pairs had Ka/Ks ratios below 1, including 41 pairs with ratios below 0.5 (**Supplementary Table 5**). These results indicated that all 42 reliably evaluated SbAP2/ERF duplicate pairs were under purifying selection.

**Figure 4:**
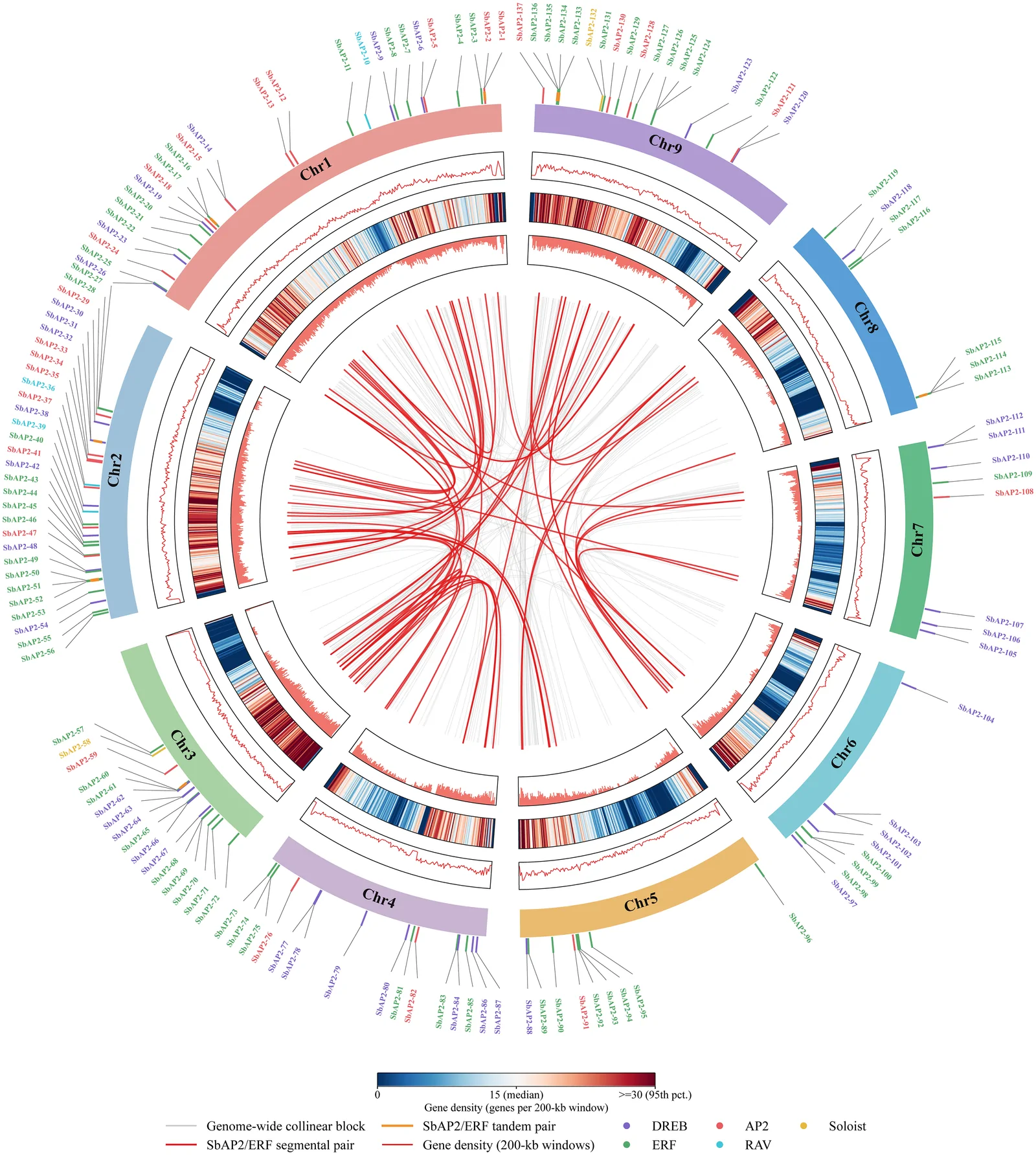
Chromosomal distribution, intragenomic collinearity, and duplication relationships of the *SbAP2/ERF* family in *S*. *baicalensis*. The colored outer sectors represent the nine chromosomes (Chr1–Chr9). The chromosomal positions of 137 *SbAP2/ERF* genes are labeled outside the sectors, and the colors of the gene names denote the AP2, DREB, ERF, RAV, and Soloist subfamilies. From outside to inside, the three tracks adjacent to the chromosome sectors show gene density as a line plot, heatmap, and histogram, respectively; all were calculated from annotated gene counts in non-overlapping 200-kb windows. The line and histogram tracks display raw gene counts per window. In the heatmap, colors from blue through white to red represent increasing gene density. Gray internal links represent 229 genome-wide collinear blocks, with one representative anchor link drawn per block. Red internal links indicate 54 *SbAP2/ERF* segmental duplicate pairs, short orange arcs outside the chromosome sectors indicate nine *SbAP2/ERF* tandem duplicate pairs.

To further investigate the evolutionary relationships of the *SbAP2/ERF* gene family across species, a collinearity analysis was performed between *S*. *baicalensis* and seven plant species, including the dicotyledons *A*. *thaliana*, *S*. *barbata*, *P*. *trichocarpa*, *S*. *lycopersicum*, *S*. *tuberosum*, and the monocotyledons *O*. *sativa*, and *Z*. *mays* (**Figure 5**). The results showed that the *SbAP2/ERF* genes exhibited 88, 124, 111, 109, 105, 43, and 42 collinear genes with *A*. *thaliana*, *S*. *barbata*, *P*. *trichocarpa*, *S*. *lycopersicum*, *S*. *tuberosum*, *O*. *sativa*, and *Z*. *mays*, respectively (**Supplementary Table 6**). The largest number of collinear gene pairs was detected between *S*. *baicalensis* and *S*. *barbata*, a pattern consistent with their close phylogenetic relationship. Additionally, *SbAP2/ERF* genes showed more collinear relationships with dicotyledonous species than with monocotyledonous species. Moreover, 21 *SbAP2/ERF* genes showed collinear relationships with all seven species, indicating conservation of the corresponding genomic regions across the sampled plant species.

**Figure 5:**
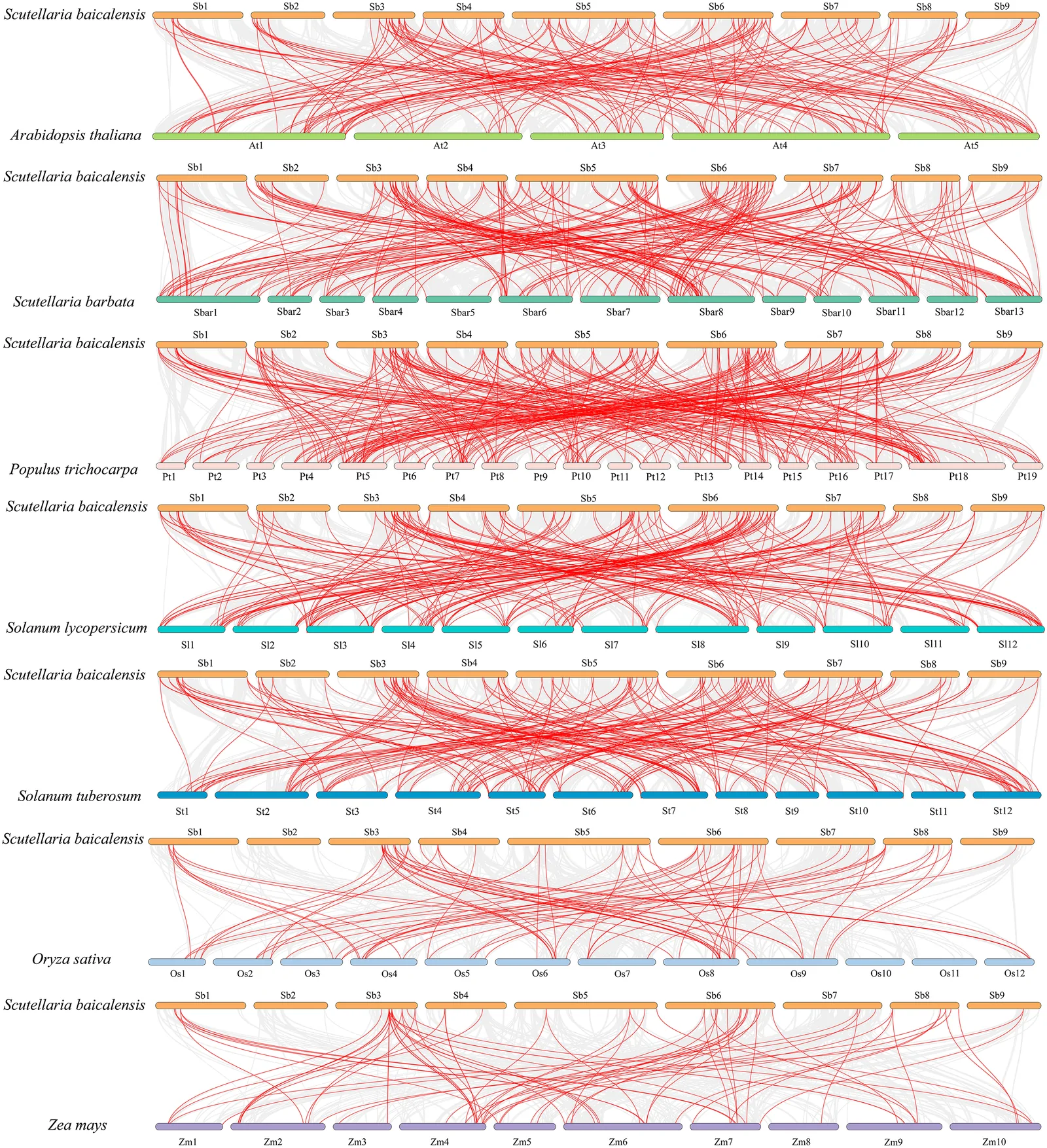
Comparative collinearity analysis of *AP2/ERF* genes between *S*. *baicalensis* and seven plant species. Collinear analyses of *AP2/ERF* genes between *S*. *baicalensis* and *A*. *thaliana*, *S*. *barbata*, *P*. *trichocarpa*, *S*. *lycopersicum*, *S*. *tuberosum*, *O*. *sativa*, and *Z*. *mays*. The gray lines between *S*. *baicalensis* and other plants represent collinear blocks in wide regions of the genomes, while red lines show the collinear relationships of *AP2/ERF* genes.

### 3.5 Analysis of cis-acting elements in the promoters of *SbAP2/ERF* members

To investigate the cis-regulatory features of *SbAP2/ERF* genes, cis-acting elements were predicted in the 2,000-bp promoter regions upstream of 137 *SbAP2/ERF* genes using the PlantCARE database. After excluding common core promoter elements such as CAAT- and TATA-boxes, 4,491 independent sites were assigned to four major functional categories: light-responsive, hormone-responsive, stress-responsive, and growth- and development-related elements (**Figure 6**; **Supplementary Table 7**). Representative elements in the four categories were shown in **Supplementary Figure 1**. Light-responsive elements were the most abundant category, with 1,801 sites (40.10%), and were present in all 137 promoters. Box 4 was the most abundant light-responsive motif, with 590 sites in 125 genes (**Supplementary Tables 7**–**9**; **Supplementary Figure 1**). Hormone-responsive elements accounted for 1,327 sites (29.55%) and were also detected in all promoters. Among them, ABRE was the most abundant, with 387 sites in 115 genes, whereas the MeJA-responsive CGTCA-motif and TGACG-motif each occurred 166 times and were jointly detected in 95 genes (**Supplementary Tables 7**–**9**; **Supplementary Figure 1**). Stress-responsive elements comprised 1,044 sites (23.25%) and were detected in all promoters, with ARE, STRE, MBS, LTR, and WUN-motif being the major motif types. Growth- and development-related elements accounted for 319 sites (7.10%) and were detected in 122 promoters, with O2-site, CAT-box, circadian, and GCN4_motif being the most frequent motifs (**Supplementary Tables 7**–**9**; **Supplementary Figure 1**). In addition, MBSI elements, annotated as MYB-binding sites associated with flavonoid biosynthesis, were identified in ten promoters (**Supplementary Table 8**).

**Figure 6:**
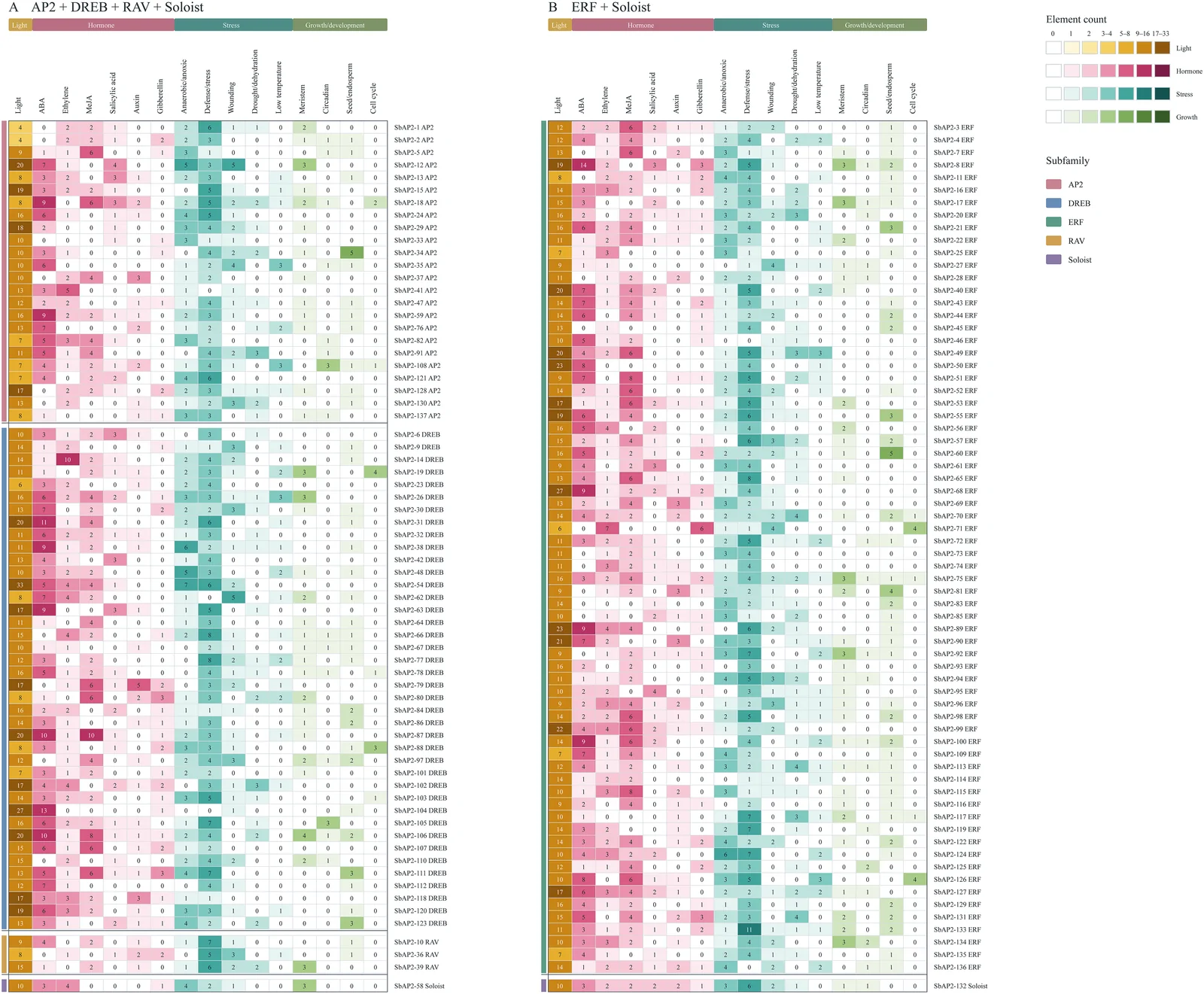
The number of predicted cis-acting elements distributed on promoter regions of *SbAP2/ERF* genes. Values indicate the number of independent predicted sites for each response type in each gene; records with the same gene, response type, position, and strand were counted once. Color intensity represents site abundance, with separate color scales for the four functional categories. Genes were grouped into the AP2, DREB, ERF, RAV, and Soloist subfamilies.

At the subfamily level, light-responsive elements remained the most abundant category after normalization by subfamily size. The AP2, DREB, and ERF subfamilies contained mean numbers of 11.25, 14.28, and 13.35 light-responsive elements per gene, respectively, followed by 8.17, 10.43, and 9.96 hormone-responsive elements per gene, respectively. The RAV subfamily showed relatively high mean numbers of light- and stress-responsive elements, whereas the Soloist subfamily displayed similar mean numbers of light-, hormone-, and stress-responsive elements (**Supplementary Figure 2**). Overall, the distribution of cis-acting elements in *SbAP2/ERF* promoters suggests broad responsiveness to light, phytohormone, and stress-related signals.

### 3.6 Expression patterns of *SbAP2/ERF* genes in different organs and under MeJA treatment

To characterize the organ expression patterns of *SbAP2/ERF* genes, two independent publicly available RNA-seq datasets from three-year-old *S*. *baicalensis* plants were analyzed. The four-organ dataset comprised roots, stems, leaves, and petals (Yao et al., 2024), whereas the three-organ dataset comprised roots, stems, and leaves (Gao et al., 2019). Mean TPM values were calculated from three biological replicates for each organ and classified into five expression ranges: low expression (TPM < 1), moderate expression (1 ≤ TPM < 10), relative high expression (10 ≤ TPM < 50), relatively higher expression (50 ≤ TPM < 100), and high expression (TPM ≥ 100). The *SbAP2/ERF* genes exhibited diverse expression patterns across the four organs shown in **Figure 7** and the three organs presented in **Supplementary Figure 3**. In the four-organ dataset, the numbers of genes assigned to these five ranges were 51, 35, 31, 9, and 11 in roots; 57, 35, 23, 6, and 16 in stems; 75, 27, 19, 6, and 10 in leaves; and 90, 34, 10, 3, and 0 in petals, respectively (**Supplementary Table 10**). In the three-organ dataset, the corresponding numbers were 58, 31, 30, 9, and 9 in roots; 56, 29, 28, 9, and 15 in stems; and 69, 27, 29, 6, and 6 in leaves, respectively (**Supplementary Table 10**). Using TPM ≥ 10 as the threshold for relative high to high expression, 51, 45, 35, and 13 genes reached this level in the roots, stems, leaves, and petals of the four-organ dataset, respectively, whereas the corresponding numbers were 48, 52, and 41 in the roots, stems, and leaves of the three-organ dataset. These results suggested that *SbAP2/ERF* transcripts were generally more abundant in vegetative organs, particularly roots and stems (**Supplementary Table 10**). Furthermore, *SbAP2-7*, *SbAP2-13*, *SbAP2-14*, *SbAP2-27*, *SbAP2-30*, *SbAP2-40*, *SbAP2-59*, *SbAP2-67*, *SbAP2-85*, *SbAP2-95*, *SbAP2-117*, *SbAP2-121*, *SbAP2-130*, *SbAP2-133*, *SbAP2-134*, and *SbAP2–137* had mean root TPM values ≥ 10 in both datasets. In each dataset, their expression levels were higher in roots than in stems and leaves, with at least one comparison reaching statistical significance (*p* adj < 0.05 and |log2FC| ≥ 1). Together, these findings suggested root-preferential expression patterns for the 16 genes (**Supplementary Table 10**).

**Figure 7:**
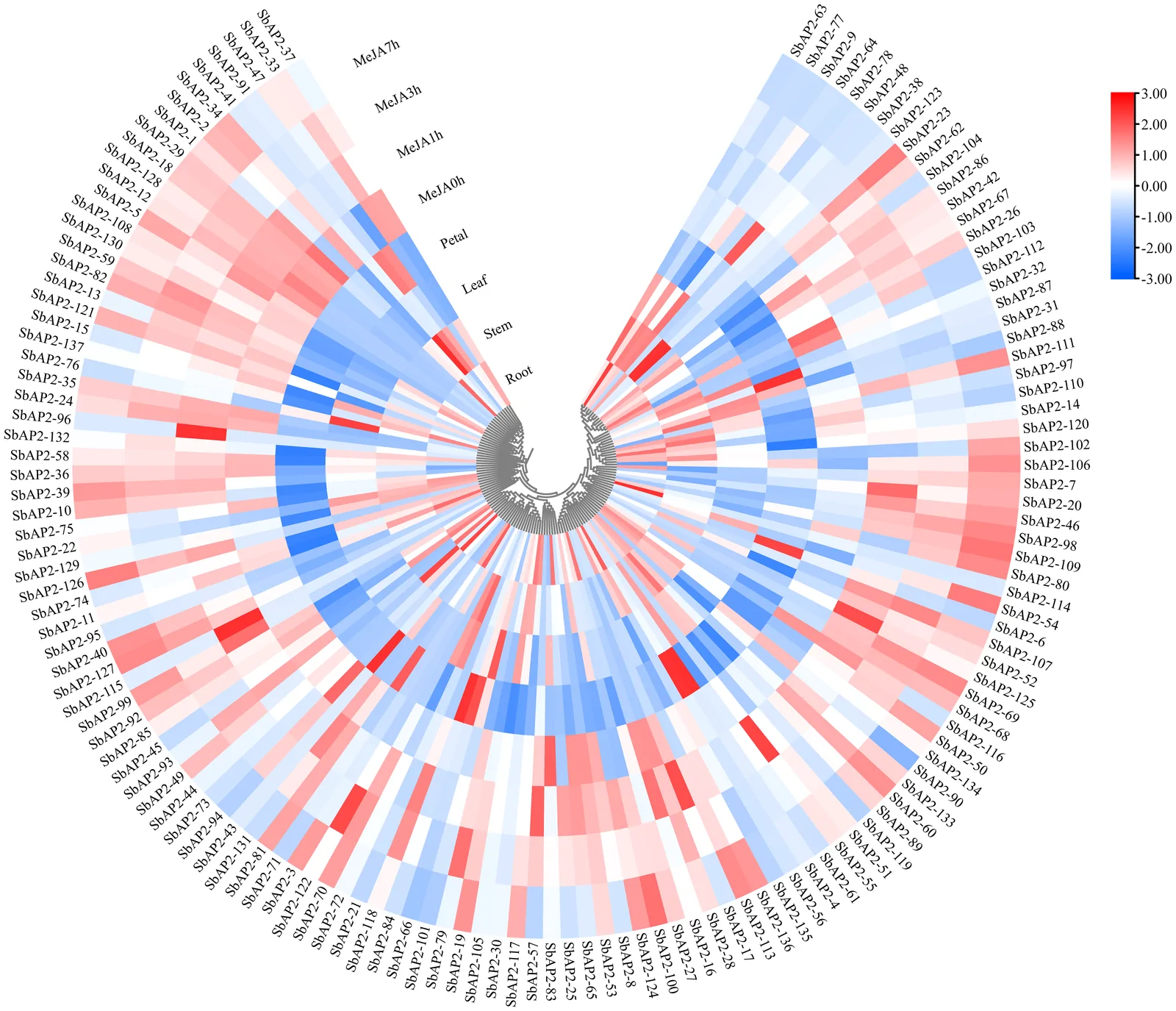
Clustered heatmaps showing the expression patterns of *SbAP2/ERF* genes across different organs and in response to MeJA treatment in *S*. *baicalensis.* Expression profiles of *SbAP2/ERF* genes across four organs (root, stem, leaf, and petal) and at different time points after MeJA treatment (0, 1, 3, and 7 h). The heatmaps were generated using log2-transformed TPM values. Colors from blue to red indicate low to high expression levels.

To investigate the responses of *SbAP2/ERF* genes to MeJA, two publicly available RNA-seq datasets derived from MeJA-treated *S*. *baicalensis* roots were analyzed. In the first dataset, roots of one-month-old plants were treated with 200 μM MeJA for 0, 1, 3, and 7 h, with three biological replicates at each time point (Fang S. et al., 2023). *SbAP2/ERF* genes showed distinct temporal expression patterns (**Figure 7**). Based on *p* adj < 0.05 and |log2FC| ≥ 1, a total of 21 *SbAP2/ERF* genes were differentially expressed at 1 h relative to the 0 h control, including 16 upregulated and 5 downregulated genes. At 3 h, 19 differentially expressed genes were identified, including 17 upregulated and 2 downregulated genes. At 7 h, the number of differentially expressed genes increased to 30, including 22 upregulated and 8 downregulated genes (**Supplementary Table 11**). Twelve *SbAP2/ERF* genes were differentially expressed at all three treatment time points. Among them, *SbAP2-23*, *SbAP2-50*, *SbAP2-52*, *SbAP2-60*, *SbAP2-62*, *SbAP2-68*, *SbAP2-69*, *SbAP2-98*, *SbAP2-100*, *SbAP2-109*, and *SbAP2–124* were consistently upregulated, whereas *SbAP2–110* was consistently downregulated. Several genes also exhibited time-dependent transcriptional responses. For example, *SbAP2–62* was significantly upregulated by 18.1-, 10.1-, and 13.9-fold at 1, 3, and 7 h, respectively. *SbAP2–3* was significantly induced only at 7 h, with a 14.7-fold increase in transcript abundance. *SbAP2–134* showed an approximately 4.0-fold increase at 1 h without reaching statistical significance but was upregulated by 3.8- and 4.4-fold at 3 and 7 h, respectively (**Supplementary Table 11**). The second dataset was obtained from roots of three-month-old *S*. *baicalensis* plants treated with 100 μM MeJA for 24 h (Zhao et al., 2019). Because biological replicates were unavailable, this dataset was used only for descriptive comparison rather than formal differential expression analysis. Based on the ratio of TPM values between MeJA-treated and untreated samples, 96 of the 137 *SbAP2/ERF* genes showed ratios greater than 1, suggesting possible relatively higher transcript abundance after MeJA treatment, whereas 38 genes showed ratios of 1 or lower, indicating unchanged or possible relatively reduced expression. The remaining three genes were not detected because of their very low expression levels (**Supplementary Table 11**). Taken together, these results demonstrated that *SbAP2/ERF* genes exhibited diverse and time-dependent transcriptional responses to MeJA treatment.

To identify candidate *SbAP2/ERF* genes with both root-preferential expression and positive responses to MeJA, the 16 root-preferential genes were further evaluated for MeJA responsiveness. In the replicated dataset, MeJA response was considered statistically supported when at least one treatment time point showed an FC ≥ 1.5 relative to the control, a *p* adj ≤ 0.05, and a mean TPM ≥ 2 in the control sample. Additionally, in the unreplicated dataset, MeJA-associated expression changes were considered descriptive evidence when they met two criteria: an FC ≥ 1.5 relative to the control and TPM ≥ 2 in the control sample. Based on these criteria, 12 genes were selected: *SbAP2-7*, *SbAP2-13*, *SbAP2-14*, *SbAP2-27*, *SbAP2-30*, *SbAP2-40*, *SbAP2-59*, *SbAP2-85*, *SbAP2-117*, *SbAP2-130*, *SbAP2-134*, and *SbAP2-137*. The organ expression patterns and MeJA responsiveness of these 12 genes were further detected by qRT-PCR (**Figures 8**, **9**). The qRT-PCR results showed that 11 genes were significantly upregulated while *SbAP2–85* was downregulated after treatment by MeJA (**Figure 8**). Among them, *SbAP2-13*, *SbAP2-14*, *SbAP2-27*, *SbAP2-40*, *SbAP2-59*, *SbAP2-85*, *SbAP2-117*, *SbAP2-130*, and *SbAP2–137* showed significantly higher expression levels in roots than in stems, leaves, and petals. By contrast, *SbAP2–7* and *SbAP2–30* showed the highest expression levels in leaves, whereas *SbAP2–134* exhibited the highest expression level in stems. Eight genes exhibited both root-preferential expression and MeJA-induced upregulation including *SbAP2-13*, *SbAP2-14*, *SbAP2-27*, *SbAP2-40*, *SbAP2-59*, *SbAP2-117*, *SbAP2-130*, and *SbAP2-137* (**Figures 8**, **9**). Considering that roots are the major organ of root-specific flavones accumulation in *S*. *baicalensis* and this accumulation could be induced by MeJA (Zhao et al., 2016b), these expression patterns may indicate a potential association of the eight genes with MeJA-responsive flavones biosynthesis in roots. Taken together, the integrated RNA-seq and qRT-PCR results highlight these eight genes as priority candidates for further functional characterization.

**Figure 8:**
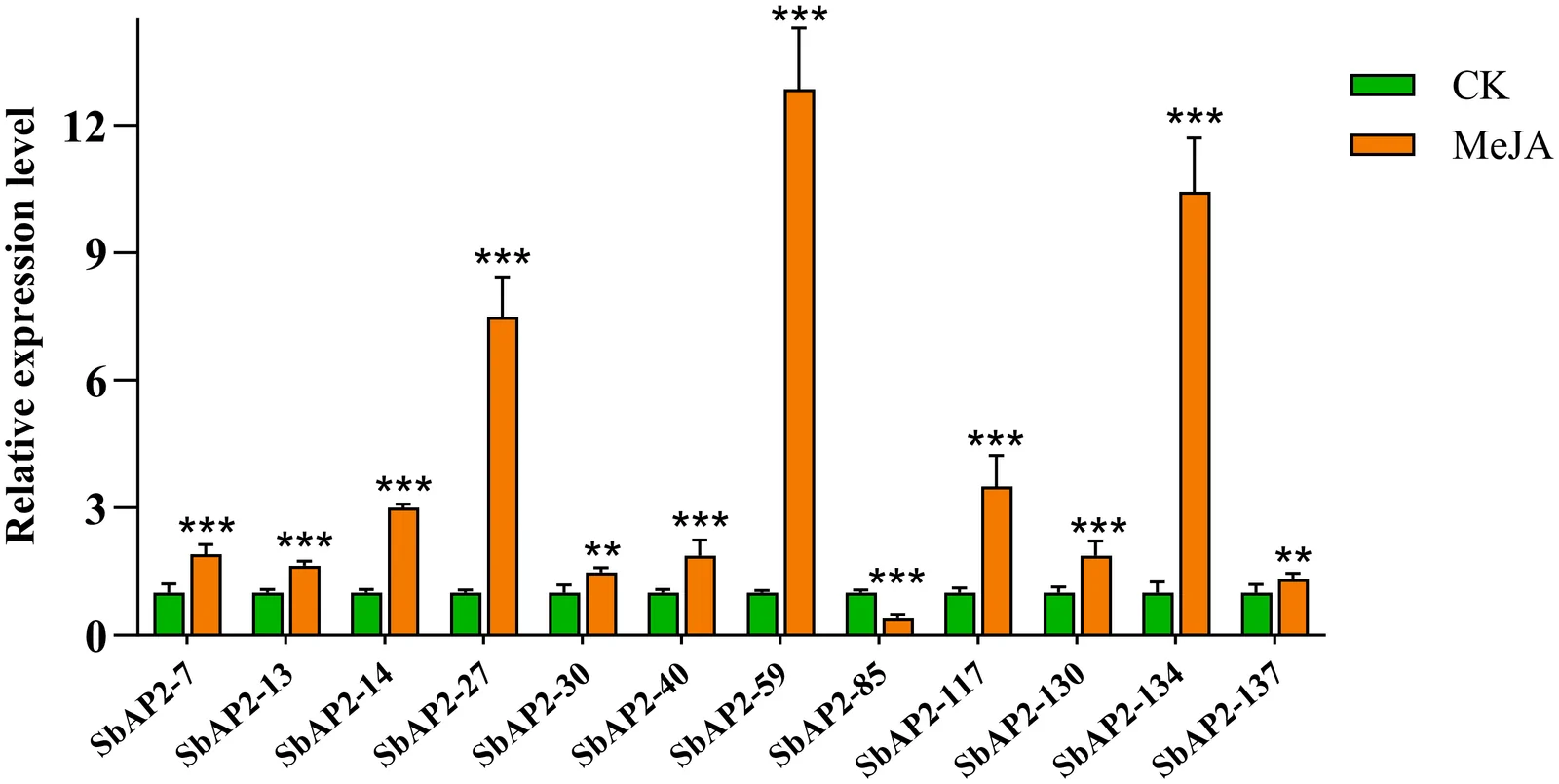
Relative expression levels of *SbAP2/ERF* genes in response to MeJA treatment in *S*. *baicalensis*. *ACT7* was used as reference gene. Statistically significant differences were indicated by ** *p* < 0.01, *** *p* < 0.001 (*t*-test).

**Figure 9:**
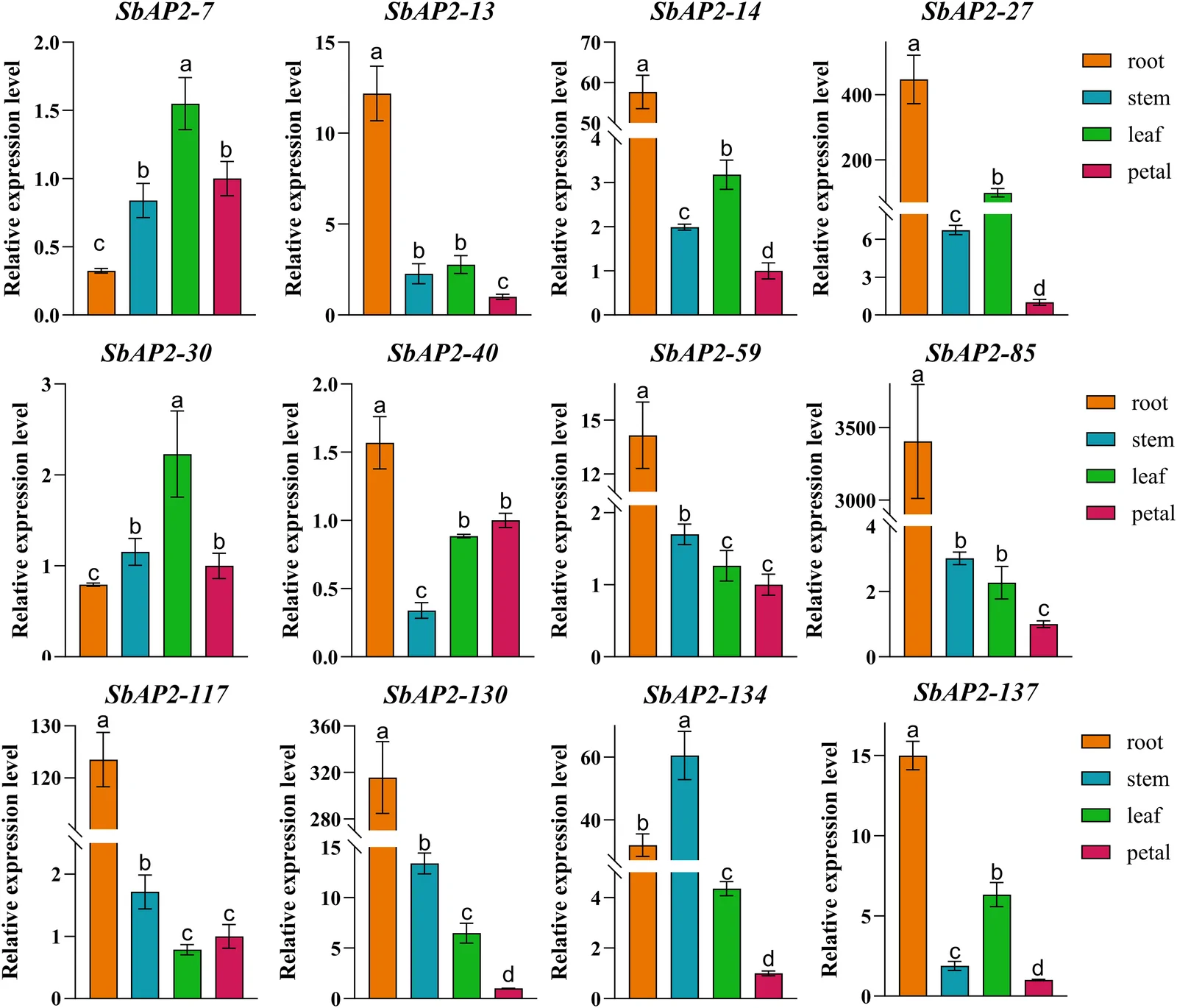
Expression patterns of *SbAP2/ERF* candidate genes in roots, stems, leaves and petals of *S*. *baicalensis*. *UBC* was used as reference gene. Different lowercase letters above the columns indicate significant differences among organs at *p* < 0.05 (Tukey’s test).

## 4 Discussion

### 4.1 Structural conservation and evolutionary diversification of the SbAP2/ERF family

The AP2/ERF superfamily is one of the largest and most functionally diverse transcription factor families in plants, with members involved in organ development, hormone signaling, stress responses, and secondary metabolism (Xie et al., 2019). In this study, 137 *SbAP2/ERF* genes were identified in the *S*. *baicalensis* genome. This number is lower than the numbers identified in *A*. *thaliana* (147) (Nakano et al., 2006), *O*. *sativa* (164) (Nakano et al., 2006), *Juglans mandshurica* (184) (Zhao et al., 2022), *Triticum aestivum* (322) (Riaz et al., 2021), *Glycine max* (301) (Wang H. et al., 2022), and *Elaeis guineensis* (172) (Zhou and Yarra, 2021), and slightly higher than that reported in *Prunus persica* (131) (Zhang et al., 2012). Differences in AP2/ERF family size among species may be associated with gene duplication, gene loss, and subsequent functional divergence (Wang et al., 2019).

Phylogenetic analysis classified the 137 SbAP2/ERF proteins into the ERF, DREB, AP2, RAV, and Soloist subfamilies. The ERF and DREB subfamilies contained 68 and 40 members, respectively, and together accounted for 78.8% of the family, whereas the AP2, RAV, and Soloist subfamilies comprised 24, 3, and 2 members, respectively (**Figure 1**). This subfamily composition is broadly consistent with the general pattern reported in *A*. *thaliana*, *O*. *sativa*, and many other plant species. ERF and DREB members, which typically contain a single AP2/ERF domain, generally constitute most of the family, whereas RAV and Soloist members are comparatively few. Most members of the AP2 subfamily contain two tandemly repeated AP2/ERF domains, and the subfamily generally contains an intermediate number of members (Nakano et al., 2006). In our study, among the 24 SbAP2 proteins, 21 displayed the typical tandem-domain architecture (**Figure 2**). Although only one confidently detected AP2/ERF domain was found in SbAP2-24, SbAP2-76, and SbAP2-137, all three gene models and CDS sequences revealed complete coding regions without obvious premature truncation, reducing the likelihood that the single domain architecture resulted from incomplete protein prediction. Moreover, moderate to strong UFBoot support for the relevant phylogenetic nodes (85%–100%), together with the consistency of their motif compositions and gene structures, provided additional support for their interpretation as provisional atypical AP2 candidates. Similar cases in which proteins containing a single AP2/ERF domain were assigned to the AP2 subfamily have also been reported in *Lactuca sativa*, *Zingiber officinale* Roscoe, *Carya illinoinensis*, and *Prunus dulcis* (Xing et al., 2021; Yang et al., 2022; Yu et al., 2022; Park et al., 2023). Gene structure and conserved motif analyses further supported this subfamily differentiation. Members of the same subfamily generally exhibited similar motif compositions and exon–intron structures, whereas clear differences were observed among subfamilies. Most *SbDREB* and *SbERF* genes contained a single exon and displayed the Motif 2–Motif 4–Motif 1–Motif 3 arrangement, whereas members of the AP2 subfamily contained 3–10 exons and exhibited more complex motif compositions (**Figure 2**). This structural pattern, characterized by greater complexity in *AP2* genes and relative simplification in *ERF/DREB* genes, has also been reported in diverse plant species, including *Brassica napus*, hybrid tea rose, and *Helianthus annuus* (Najafi et al., 2018; Ghorbani et al., 2020; Yan et al., 2024). Streamlined gene structures have been proposed to reduce the time required for transcription and RNA processing, potentially facilitating rapid responses to environmental stress (Speth et al., 2018). Accordingly, the relatively simple gene structures of the SbDREB and SbERF subfamilies may be compatible with their capacity to respond rapidly to environmental changes. In contrast, the SbAP2 subfamily has retained more complex gene structures which might be associated with its more elaborate developmental regulatory functions and the need for stricter spatiotemporal control of gene expression (Xie et al., 2019). Overall, SbAP2/ERF members show structural similarity within individual subfamilies but clear structural differentiation among subfamilies, which might contribute to their functional diversification.

Cross-species collinearity analysis revealed that a subset of *SbAP2/ERF* genes retained collinear relationships across the sampled angiosperms, although the extent of collinear conservation varied among species comparisons. The largest number of collinear gene pairs was detected between *S*. *baicalensis* and *S*. *barbata* (**Figure 5**), consistent with their placement within the same genus and their relatively close phylogenetic relationship. Previous comparative genomic analysis also identified extensive collinear relationships between orthologous genes in *S*. *baicalensis* and *S*. *barbata* (Xu et al., 2020). More SbAP2/ERF-related collinear relationships were generally detected with the sampled dicotyledonous species than with *O*. *sativa* and *Z*. *mays.* This pattern is compatible with the early divergence between monocotyledonous and dicotyledonous lineages. The collinearity analyses across species and the duplication analyses within the genome revealed the coexistence of expansion in specific lineages and evolutionary retention within the SbAP2/ERF family. Among the 63 nonredundant duplicated gene pairs identified in this study, 54 resulted from segmental duplication, whereas nine were attributed to tandem duplication (**Figure 4**), indicating that segmental duplication was probably the predominant mode of SbAP2/ERF family expansion. This pattern is consistent with findings reported for WRKY and R2R3-MYB families in *S*. *baicalensis* (Wang et al., 2022b; Zhang et al., 2022), which suggested that segmental duplication may be mainly responsible for the expansion of multiple transcription factor families in *S*. *baicalensis*. In this study, all 42 duplicate gene pairs with valid Ka/Ks estimates had Ka/Ks ratios below 1, with 41 exhibiting ratios below 0.5 (**Supplementary Table 5**). Notably, 15 of the 21 *SbAP2/ERF* genes that retained collinearity counterparts across all seven comparison species also appeared among these 42 duplicate pairs, involving 15 pairs in total (**Figure 5**; **Supplementary Table 6**). Together, these findings indicated that some *SbAP2/ERF* genes with collinearity retained across species also participated in intragenomic duplication in *S*. *baicalensis*, and these genes were generally subject to purifying selection following duplication, suggesting that both their retention within the genome and their protein sequences were highly conserved during evolution. By contrast, SbAP2-1, SbAP2-3, and SbAP2–5 showed detectable collinear relationships only with *S*. *barbata* among the seven comparison species (**Figure 5**). This restricted pattern might reflect retention shared by the two sampled *Scutellaria* species, loss of homologous chromosomal regions in the other sampled lineages, or extensive sequence and structural divergence.

### 4.2 MeJA responsiveness and identification of root-preferential candidate *SbAP2/ERF* genes

Promoter cis-acting element analysis provided clues to the potential upstream regulatory signals of *SbAP2/ERF* genes. After excluding common core promoter elements such as the CAAT-box and TATA-box, a total of 4,491 deduplicated independent functional sites were identified within the 2,000-bp upstream sequences of the 137 *SbAP2/ERF* genes. Light-responsive, hormone-responsive, and stress-responsive elements accounted for 40.10%, 29.55%, and 23.25% of these sites, respectively, and were widely distributed across the *SbAP2/ERF* promoters (**Supplementary Tables 7**, **8**). Hormone- and stress-related elements have also been widely identified in the promoters of *AP2/ERF* genes in other plant species, including *Prunus dulcis* and *Passiflora edulis* (Luo et al., 2025; Zhang et al., 2025). Together, these promoter features suggested that the transcription of *SbAP2/ERF* genes might be responsive to multiple environmental and endogenous signals, including those related to light, phytohormones, and stress.

Notably, MeJA-responsive elements were widely distributed across *SbAP2/ERF* promoters. The CGTCA-motif and TGACG-motif each occurred at 166 sites and co-occurred in the promoters of 95 *SbAP2/ERF* genes (**Supplementary Table 8**), suggesting that MeJA represents a prominent candidate upstream signal influencing the transcriptional responses of this family. MeJA is an important signaling molecule involved in the regulation of plant defense responses and secondary metabolism and has been closely associated with the accumulation of bioactive constituents in several medicinal plants, including *Ginkgo biloba*, *Panax notoginseng*, and *Murraya koenigii* (Tan et al., 2021; Singh et al., 2023; Dai et al., 2024). It has been reported that MeJA treatment increased the accumulation of root-specific flavones in hairy root cultures of *S*. *baicalensis* such as baicalin, baicalein, and wogonin (Zhao et al., 2016b). And various transcription factor families mediate the complex regulation of jasmonic acid responses in plants, including AP2/ERF, MYB, bHLH, and WRKY (Fang S. et al., 2023; Liu H. et al., 2023; Yuan et al., 2023; Huang et al., 2024). Several MYB transcription factors in *S*. *baicalensis* have been shown to directly regulate structural genes involved in flavone biosynthesis. SbMYB3 binds to and activates the *SbFNSII-2* promoter, thereby affecting the accumulation of baicalin, baicalein, wogonoside, and wogonin. SbMYB12 binds to the promoters of *SbCCL7-4*, *SbCHI-2*, and *SbF6H-1* and promotes baicalin biosynthesis (Wang et al., 2022a; Fang Y. et al., 2023). In our study, MBSI elements were detected in the promoters of ten *SbAP2/ERF* genes (**Supplementary Tables 8**, **9**), suggesting that a subset of *SbAP2/ERF* genes might be subject to MYB-associated upstream regulation. These predicted elements provide clues at the sequence level regarding potential transcriptional regulatory connections between MYB and AP2/ERF factors in *S*. *baicalensis*. Furthermore, several *AP2/ERF* genes in *Eschscholzia californica* and *Panax ginseng* exhibit MeJA-induced expression, indicating that responsiveness of AP2/ERF members to jasmonate signaling is relatively common among different medicinal plant species (Chen et al., 2020; Yamada et al., 2020).

Consistent with the potential MeJA responsiveness predicted by promoter analysis, RNA-seq data from MeJA-treated *S*. *baicalensis* roots revealed temporal changes in *SbAP2/ERF* transcript abundance. Individual genes differed in both the magnitude and timing of their responses. *SbAP2–62* increased rapidly by 18.1-fold at 1 h and remained at relatively high levels at 3 and 7 h, with increases of 10.1- and 13.9-fold, respectively, indicating a rapid and sustained response pattern. In contrast, *SbAP2–3* showed its strongest increase at 7 h, when its transcript abundance increased by 14.7-fold, indicating a relatively late response. *SbAP2–134* increased by 3.8- and 4.4-fold at 3 and 7 h, respectively (**Supplementary Table 11**). These temporal differences suggested that individual *SbAP2/ERF* members may participate in different phases of the MeJA response.

Expression patterns among organs may reflect functional diversification within the SbAP2/ERF family. AP2 members are commonly associated with organogenesis and developmental regulation, DREB members with environmental response pathways, and ERF members with hormone related and defense related transcriptional networks (Xu et al., 2013; Lee et al., 2016; Liu X. et al., 2023; Wang et al., 2024; Tang et al., 2025). In addition, individual AP2/ERF members have been reported to regulate specialized metabolism in a manner that varies among species and genes. Therefore, the higher transcript abundance of *SbAP2/ERF* genes in roots and stems than in petals may reflect the combined contributions of genes involved in root/stem development, hormone signaling, defense responses, and specialized metabolism. Additionally, root is the principal medicinal organ of *S*. *baicalensis* and an important site of accumulation of root-specific flavones such as baicalin, baicalein, wogonoside, and wogonin (Pei et al., 2022). Previous studies have shown that several genes involved in this pathway exhibit root-preferential expression. Among roots, stems, leaves, and flowers, *SbCLL-7*, *SbCHS-2*, *SbFNSII-2*, *SbF8H* were reported to show their highest transcript levels in roots. Moreover, *SbCHS-2* and *SbFNSII-2* were further induced by MeJA treatment (Zhao et al., 2016b, 2018). These published findings indicate that root-preferential expression and MeJA responsiveness are potential characteristic features of genes involved in root associated flavone biosynthesis. Therefore, the overlap between relatively high root expression and MeJA-responsive transcription provided both spatial and treatment related evidence for selecting SbAP2/ERF members as candidates for further investigation of MeJA responsive processes and their potential association with root-specific flavones biosynthesis in *S. baicalensis*.

By integrating RNA-seq and qRT-PCR evidence, eight candidate genes were identified including *SbAP2-13*, *SbAP2-14*, *SbAP2-27*, *SbAP2-40*, *SbAP2-59*, *SbAP2-117*, *SbAP2-130*, and *SbAP2-137*. All eight candidates were significantly upregulated by MeJA and were expressed at significantly higher levels in roots than in stems, leaves, and petals (**Figures 8**, **9**). Additionally, the MeJA-responsive cis-acting elements were predicted in the promoters of *SbAP2-14*, *SbAP2–40* and *SbAP2–59* further providing clues at the sequence level regarding their possible responsiveness to MeJA (**Supplementary Table 9**). These candidates provide targets for further investigation of the transcriptional regulatory network underlying MeJA responses in *S*. *baicalensis* roots and its potential connections with root-specific flavones biosynthesis.

## 5 Conclusion

In this study, 137 *AP2/ERF* family genes were systematically identified in the *S*. *baicalensis* genome and classified into five subfamilies: ERF, DREB, AP2, RAV, and Soloist. Analyses of conserved motifs, domain architectures, gene structures, chromosomal distribution, gene duplication, and interspecific collinearity revealed clear conservation and evolutionary divergence within this family. Cis-acting elements of light-, hormone-, and stress-responsive were widely distributed in the promoters of *SbAP2/ERF* genes. Analysis of publicly available RNA-seq datasets showed that *SbAP2/ERF* genes exhibited diverse organ expression patterns and time-dependent transcriptional responses to MeJA. By integrating evidence of RNA-seq and qRT-PCR validation, eight candidate genes exhibiting both root-preferential expression and significant MeJA induction were identified including *SbAP2-13*, *SbAP2-14*, *SbAP2-27*, *SbAP2-40*, *SbAP2-59*, *SbAP2-117*, *SbAP2-130*, and *SbAP2-137*. Overall, this study not only provided a systematic resource for understanding the evolution and transcriptional characteristics of the SbAP2/ERF family but also identified candidates for further elucidating MeJA-responsive processes and the potential transcriptional regulation of root-specific flavones biosynthesis in *S*. *baicalensis*.

## Data Availability

The original contributions presented in the study are included in the article/Supplementary Material. Further inquiries can be directed to the corresponding authors.

## Supplementary material

Supplementary Figure 1 Positional distribution of representative cis-acting elements in the promoters of 137 *SbAP2/ERF* genes. Five elements were selected from each functional category based on abundance, gene coverage, or functional representativeness. Colored rectangles indicate element positions along the 2,000-bp region upstream of the transcription start site; the left and right ends correspond to −2,000 bp and 0/TSS, respectively. G-box and G-Box are displayed as G-box-related, and CGTCA-motif and TGACG-motif are displayed as MeJA-responsive.

Supplementary Figure 2 Mean numbers of four functional cis-acting element categories in promoters of different *SbAP2/ERF* subfamilies. Bars indicate the mean number of independent predicted sites per gene in each subfamily. Sites were deduplicated by gene, functional category, promoter position, and strand. Light-responsive, phytohormone-responsive, stress-responsive, and growth- and development-related elements are shown in different colors.

Supplementary Figure 3 Clustered heatmaps showing the expression patterns of *SbAP2/ERF* genes across different organs in *S*. *baicalensis.* Expression profiles of *SbAP2/ERF* genes across three organs (root, stem, leaf). The heatmaps were generated using log2-transformed TPM values. Colors from blue to red indicate low to high expression levels.
